# SWAT-based soil erosion and sediment yield modeling in the upper Gilgel Abay catchment, Blue Nile Basin, Ethiopia

**DOI:** 10.1038/s41598-025-09367-3

**Published:** 2025-08-06

**Authors:** Endalamaw Dessie Alebachew, Kebede Wolka, Mikias Biazen Molla, Nega Chalie Emiru, Orhan Dengiz, David Tavi Agbor

**Affiliations:** 1 Hawassa University, Wondo Genet College of Forestry and Natural Resources, Hawassa, Ethiopia; 2 Faculity of Agriculture, Department of Soil Science and Plant Nutrition, Ondokuz Mayıs University, Samsun, Turkey

**Keywords:** Catchment, Sediment, Soil Erosion modelling, SWAT, Environmental sciences, Hydrology

## Abstract

Soil erosion is a critical global issue, threatening the sustainability of natural resources and agricultural productivity. Accurate information on sediment yield and soil erosion risk within watersheds is essential for developing effective management strategies. This study aimed to estimate soil loss in the Upper Gilgel Abay catchment using the Soil and Water Assessment Tool (SWAT). The observed daily sediment data were utilized for the calibration and validation of the model outputs. Calibration and validation were performed for the periods 2002–2015 and 2016–2021, respectively. Sensitivity analysis identified the most influential parameters affecting soil loss estimates. The model performance was quite satisfactory. The coefficient of determination (R^2^) was 0.69 for the calibration and 0.67 for the validation of sediment yield. Over the past 18 years, the Gilgel Abay catchment experienced approximately 1.5 billion tons of soil loss, with spatial analysis revealing soil loss rates of up to 53.88 t/ha/year, particularly in the upper catchment. The maximum and minimum annual sediment yields at the outlet of the catchment were 318, 233 tons, and 61,575 tons per year, respectively. On average 184,695 tons of soil leave the catchment annually. While the model provided an acceptable level of the average basin-wide soil loss, certain areas exhibited severe erosion. These findings underscore the urgent need for targeted soil conservation practices, such as terracing, reforestation, and sustainable land management, to mitigate soil degradation and enhance catchment sustainability.

## Introduction

According to the Global Soil Partnership hosted by FAO, arable land loses 75 billion tons of soil each year, which is equal to a USD 400 billion loss in the economy^1^. Approximately 36 billion tons of soil is lost worldwide each year, as per the latest estimates^2^. The rate of soil erosion in the world is between 12 and 15 t ha^-1^ y^-1^^3^. It shows that the removal of the top layer 0.96 to 1.2 mm annually, resulting in losses of between US$64 and US$80 billion^4^. Soil erosion accelerates the occurrence of land degradation in tropical regions such as the Ethiopian highlands. It has a negative consequence for food security, agricultural productivity and sustainable management of natural resources^5,6^.

Ethiopia has extremely high rates of soil erosion, with the highlands losing roughly 3.5 billion tons of soil per year, 1.5 billion of which depart the nation through its rivers. Ethiopia’s environmental issue is grave and ongoing due to soil erosion. Due to Ethiopia’s uneven topography, soil erosion is a major environmental problem that has a substantial effect on agricultural productivity and the long-term viability of those areas^7,8^. The critical nature of this problem has been revealed by recent investigations. For instance, a synthesis of modeling and plot-level research by Tadesse et al.^9^ demonstrated that assessments of soil erosion in Ethiopia have attracted a lot of interest in the past 40 years. The study underscored the importance of boosting watershed and plot-level monitoring, especially in arid and semi-arid regions. Furthermore, the lower Baro watershed’s shifting land cover a reduction in wetlands, shrubland, rangeland, and forest land, and an increase in built-up regions, agricultural land, and water bodies have had a substantial impact on hydrological variables^10^.

Sedimentation in downstream or sediment deposition in the reservoirs is a serious off-site impact of soil erosion as it shortens the reservoir’s design life and reduces the dams’ sustainability^11^. Sediments are frequently seen as an unfavorable but unavoidable side effect of water storage. Reservoir sedimentation results in an annual loss of 2% of the intended storage volume^12^. In addition to affecting the quality of water bodies, the erosion of the catchments’ most fertile soils reduces their capacity to hold water through the sedimentation process^13^.

Water-induced soil erosion continues to be the primary driver of land degradation, making it the most significant environmental issue in the world. Additionally, a lack of appropriate erosion prevention measures and an increase in farming activities are major contributors to this issue^14,15^. To estimate runoff-induced soil erosion, soil erosion models are frequently used worldwide, such as the Universal Soil Loss Equation (USLE) and its updated version Revised Universal Soil Loss Equation (RUSLE), the Soil and Water Assessment Tool (SWAT), the European Soil Erosion Model (Euro SEM) the Water Erosion Prediction Project (WEPP)^7^. The updated version of the Universal Soil Loss Equation (RUSLE) has limitations, including the result obtained using this model mainly depending on the input data given by the expert during processing, which could be affected by personal bias and error^16^. Accelerated erosion via sheet and rill erosion results in a deposition in lake Tana^17^. Globally, the SWAT model has been effectively used for determining the extent to which Soil and Water Conservation (SWC) methods reduce runoff, reduce soil erosion, and increase groundwater recharge^18^. The SWAT model effectively simulates hydrological and sediment processes allowing for the identification of erosion hotspots and application of effective conservation measures^19^. Studies conducted in the Hombole, Weyb and Gidabo, watersheds have validated SWAT’s performance in quantifying soil erosion and sediment yield^20,21^. In Ethiopia, research combining the Soil and Water Assessment Tool (SWAT) with geospatial technologies in various watersheds has emphasized the critical role of vegetation covers and proper land-use management in reducing soil erosion^22^. According to Gebresamuel et al.^23^, these models have been used in studies carried out in similar watersheds, like the Chamo basin in Southern Ethiopia. Furthermore, a study by Leta et al.^24^ utilized the SWAT model to estimate sediment yield and soil erosion in the Nashe watershed, Ethiopia. This research identified key areas contributing to sediment and assessed the total amount of soil loss in the watershed. In this case, identifying the priority locations for intervention and assessing the effectiveness of soil conservation measures have been made possible by the application of the SWAT model.

The upper Gilgel Abay catchment faces a land degradation problem with soil erosion from upstream and subsequent sedimentation in the downstream area. Due to its complicated terrain, the upper Gilgel Abay catchment is particularly vulnerable to soil erosion, which could be exacerbated by several factors such as high rates of wind and water erosion, overgrazing, high rainfall variability, high rainfall intensity, and heavy rainfall^25^. Even though this issue is critical, prior research has mostly concentrated on general evaluations of soil erosion in the area, paying little thought to the regional variations in erosion risk and sedimentation mechanisms. Prior research has primarily used empirical models to conduct generalized assessments of soil erosion. For instance, Bewket and Teferi^26^ used remote sensing data to perform a qualitative assessment of land degradation based on changes in land use and land cover, while Gelagay and Minale^27^ used the Revised Universal Soil Loss Equation (RUSLE) combined with GIS and satellite imagery to estimate annual soil loss around Koga watershed. Despite their value, these studies frequently lack spatially explicit modeling of the dynamics of sediment yield and routing, particularly at the catchment outlet. As a result, the accurate determination of the risk of soil erosion and the amount of sediment at the outlet is essential for sustainable watershed management. Estimation of soil loss effectively and conveniently, evaluating the risk of soil erosion, and applying conservation measures become an urgent task. This study attempts to fill this gap by using high-resolution remote sensing data and the SWAT model to determine the risk of soil erosion, calculate the amount of sediment deposited at the outlet, and locate the Upper Gilgel Abay catchment’s priority areas for conservation.

## Materials and methods

### Study area description

**Fig. 1:**
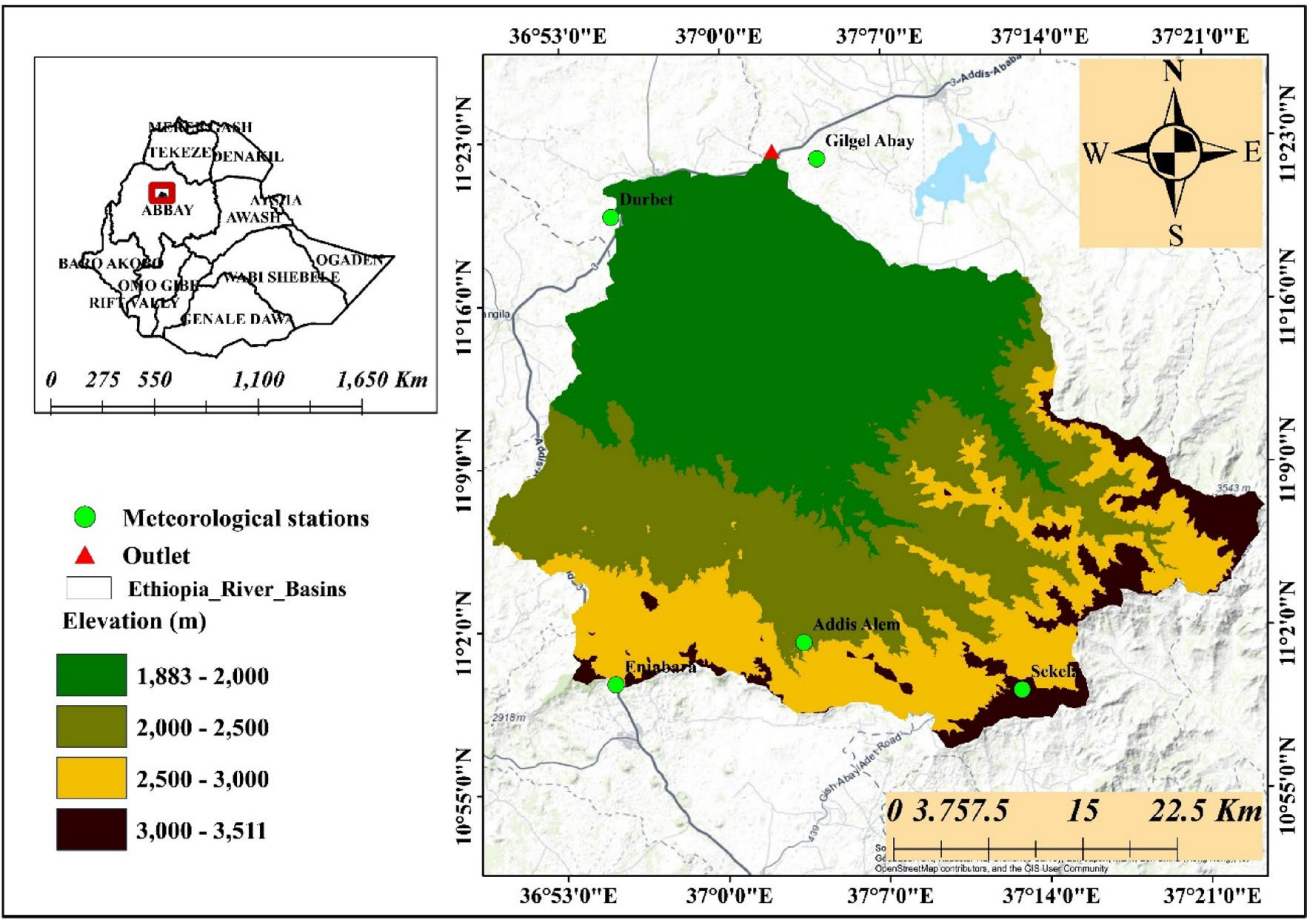
Location map of the study area. The map was produced using ArcGIS (Version 10.8.2, https://www.esri.com/en-us/arcgis/products/arcgis10.8.2).

The upper Gilgel Abay catchment, which corresponds to the gauged portion of the Gilgel Abay river basin, is one of the major tributaries of Ethiopia’s lake Tana basin. It encompasses a total area of 163,557.31 ha. It is located at 10° 56′ 53″ to 11° 21′ 58″N latitudes and 36° 49′ 29″ to 37° 23′ 34″E longitudes^17,28^ (Fig. 1).

The southern and southeastern regions of the catchment consist of highlands with mountain ranges, whereas the northern edge, despite being at a high altitude, is relatively flat compared to the rest of the area^29^. The elevation varies from 1883 m a.s.l. to 3511 m a.s.l. The mean annual precipitation in the Upper Gilgel Abay catchment is approximately 1400 mm and the mean daily minimum and maximum temperatures range between 15 °C and 23 °C on average^17,30^. Andosols, Luvisols, and Vertisols are the three main soil orders in the catchment^31^.

The natural vegetation in the Upper Gilgel Abay catchment is represented by Cordia africana, Juniperus procera, Croton macrostachyus, Rumex nervosus Vahl, Carissa spinarum, Dodonaea angustifolia, Olea europaea, and Rosa abyssinica Lindley^32^. Cropland dominates the Gilgel Abay catchment, with a few scattered forests and forested peaks. Grassland, marshes, and woodland with regular areas of shrubs and eucalyptus forests are the predominant land cover types, aside from cultivated fields^28^.

### The SWAT model

SWAT is a hydrological model developed by the USDA to predict how different land management practices will affect agricultural productivity^33^. The model utilizes spatial data from geospatial technologies, incorporating an integrated physical framework that considers different factors such as climate, topography, soil properties and land use and management practices^34^. The SWAT model, selected for its flexibility and strong performance in hydrological studies, has been widely utilized to simulate runoff, evaluate the effects of climate and land-use changes, and aid in water resource management across various environmental and agro-climatic settings^35^. During modeling, the catchment was separated into several sub-catchments to reduce the running time and help get accurate results^36^. The water balance equation was applied to determine the hydrological process occurring in the catchment Eq. (1)^33^.

1 $$\:{SW}_{0}={SW}_{1}+(R-{Q}_{surf}-E\alpha\:-{W}_{seeg}-{Q}_{gd}).$$

Where SW_1_ is the final water content of the soil (mm), SW_0_ is the soil initial soil water content on day i (mm), E_α_ is the evapotranspiration on a day i (mm), R is the precipitation on day i (mm), W_seeg_ is the water entering from the soil profile on a day i (mm), Q_surf_ the surface runoff on day i (mm), and Q_gd_ is the return flow on a day i (mm)^37^.

Soil erosion and sediment yield was estimated using a modified version of the universal soil loss equation (MUSLE). Modified Universal Soil Loss Equation (MUSLE) predict sediment yield as a function of runoff factor on a given day^38^. MUSLE is applied in SWAT by assuming a basic hydrograph shape to estimate daily runoff volume and peak flow rate within each sub-watershed, which is then used to predict changes in runoff erosive energy. SWAT calculates sediment yield as total sediment load and as fractions of sand, silt, and clay for each sub-watershed Eq. (2)^39^.

2 $$\:A=11.8\:({Q}_{surf}\times\:{q}_{peak}\times\:{A}_{hru}{)}^{0.56}\times\:K\times\:LS\times\:C\times\:P\times\:{C}_{frg}.$$

Where A represents the sediment yield (metric tons), Q_surf_ the surface runoff (mm per hectare), q_peak_ the peak rate of runoff (m3/s), A_hru_ the hydrologic response unit area (hectares), K the soil erodibility factor, C the cover and management factor, P the support practice factor, LS the slope length and steepness factor, and C_frg_ the coarse fragment^40^.

The SWAT model was selected as an effective model for forecasting soil erosion and sediment yield for the following reasons^41^. SWAT considers the entire water system of the catchment while RUSLE focuses on estimating soil erosion on individual plots. It considers the spatial variability of the physio-chemical properties of the soil and the land use activities practiced in the area allowing for a comprehensive analysis of water resources and sediment yield. It considers the complexity of the catchment and relies on the detailed knowledge of climate data, soil properties as well as vegetation cover in the catchment. It is computationally efficient, easily accessible and unrestricted^42^. SWAT is used in a wide range of Ethiopian scenarios (suitable and yielded positive results)^33^.

### SWAT model input data and sources

The catchment boundary and catchment slope were created using SRTM 1 Arc-Second Global DEM (30 m) obtained from the USGS website (Table 1). The SRTM 1 Arc-Second Global DEM is a crucial input for the SWAT model, providing essential information about the topography of the watershed, including stream network delineation, watershed boundary definition, slope, and flow direction. Daily climatic data were obtained from the National Meteorological Institute of Ethiopia. Soil data were obtained from the ISRIC World Soil Information database and used to prepare a soil map of the catchment. World imagery from 2020 with a 1.19 m resolution, accessed through ArcGIS Pro, was used to prepare a land use map of the area. Measured hydrological data were obtained from the Ministry of Water and Energy, Ethiopia (MoWE).

**Table 1:** Data types and sources.

Data types	Sources	Resolution	Temporal scale	Description/purposes
SRTM 1 Arc-Second Global DEM	USGS. https://earthexplorer.usgs.gov/	30 m	–	To generate catchment boundary and slope of the watershed
Soil data	ISRIC World Soil Information, Africa Soil Profiles Database	250 m	–	To generate a soil map
Weather data	National Meteorological Institute, Ethiopia	–	2001–2021	To produce surface runoff and potential evapotranspiration
Land use	World imagery in ArcGIS Pro	1.9 m	2021	To determine how the landscape interacts with water and sediment.
Measured stream flow and sediment data	Ministry of Water and Energy, Ethiopia	–	2002–2021	Calibration and validation of the simulated output [45]

#### Weather

One of the major inputs used by the SWAT Model is climate data. The daily climatic data required by SWAT for simulation were obtained from the National Meteorological Institute of Ethiopia. The climatic data were taken from four meteorological stations, including Sekela, Durbet, Gilgel Abay and Adis Alem stations. Each station provided data on precipitation, as well as maximum and minimum temperatures, as shown in Table 2. The record period spans from January 2001 to December 2021, except for the Sekela station, which covers 2001–2018, with missing data from 2019 to 2021 filled using CHIRPS. Climate Hazards Group InfraRed Precipitation with Station data (CHIRPS) is a high-resolution rainfall dataset developed by the Climate Hazards Group at the University of California, Santa Barbara. It integrates ground-based station data with satellite imagery to generate reliable precipitation estimates. CHIRPS offers quasi-global coverage (50°S–50°N) from 1981 to the present, with a spatial resolution of 0.05° (~ 5 km) and temporal resolutions of daily, pentadal, and monthly. It is particularly useful in regions with limited ground data and is freely available at (https://www.chc.ucsb.edu/data/chirps). According to Bennett^43^, The CHIRPS dataset, derived from rain gauge measurements and satellite observations, demonstrated the highest performance among the open-access precipitation products reviewed, as evaluated using scatterplots and correlation coefficients. This included comparisons with products such as CFSR and ERA5. CHIRPS was also employed to address missing values in some climatic variables at individual stations. Before integration into the model, the weather data underwent quality control procedures, including the detection of missing or inconsistent values. In cases of data gaps, CHIRPS precipitation data was specifically employed to fill missing records for individual stations, ensuring the continuity and reliability of the weather dataset for the simulation. The final step in handling weather data involved formatting all climate datasets into SWAT-compatible input files (i.e., text files).

**Table 2:** Meteorological stations and the availability of Climatic data.

Station name	Long (deg)	Lat (deg)	Year	Data type					
				Precipitation	Max. temperature	Min. temperature	Wind speed	Solar radiation	Relative humidity
DURB	37.43	10.99	2001–2021	✓	✓	✓			
SEKL	37.21	10.98	2001–2018	✓	✓	✓		✓	✓
GLGA	37.04	11.37	2001–2021	✓	✓	✓			
ADAL	37.05	11.02	2001–2021	✓	✓	✓			

#### Soil data

**Fig. 2:**
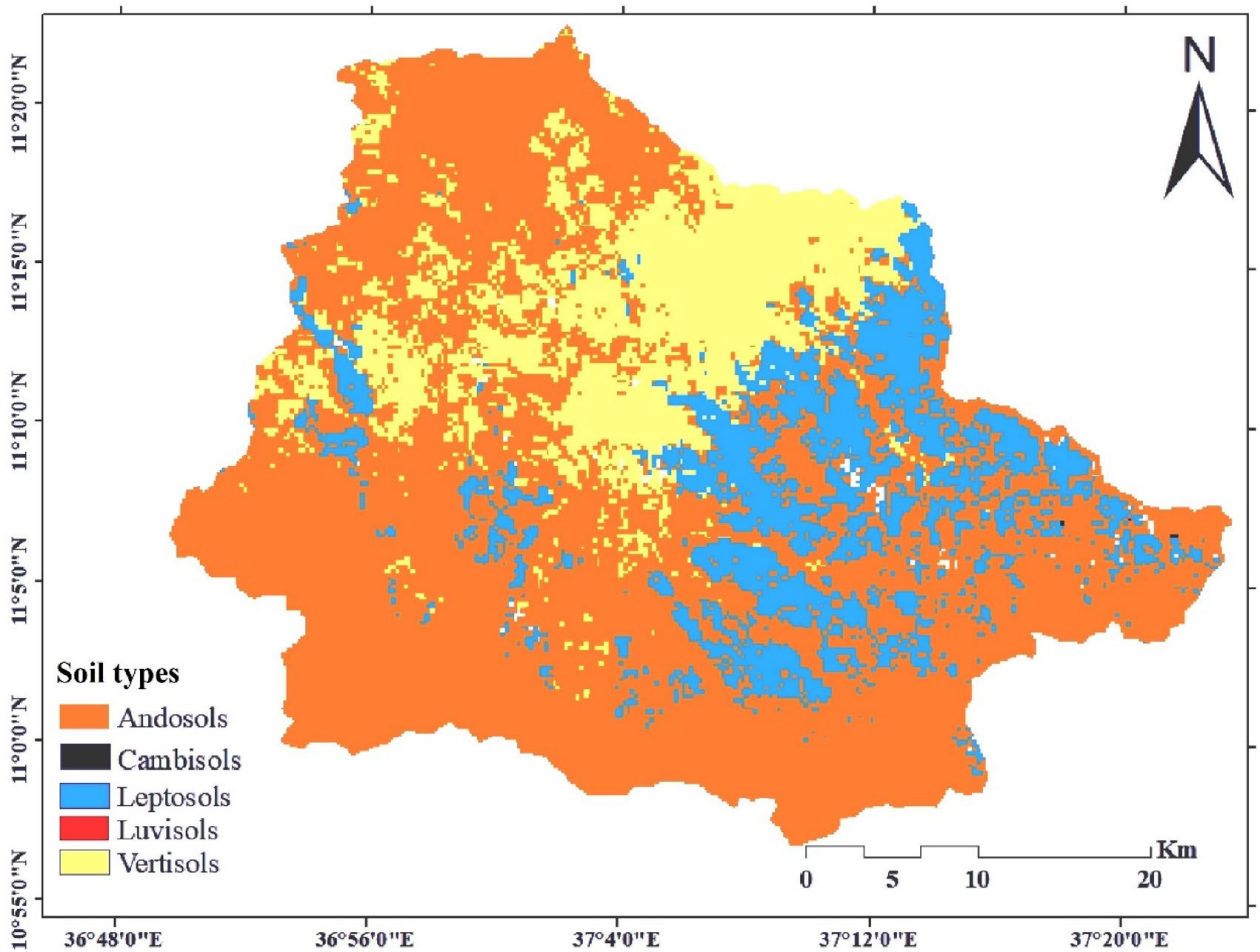
Soil map. The map was produced using ArcGIS (Version 10.8.2, https://www.esri.com/en-us/arcgis/products/arcgis10.8.2).

Soil data including soil physio-chemical properties is also one of the key inputs of the SWAT model. Five significant soil groups were found in the catchment using the FAO/UNESCO - ISRIC categorization (Fig. 2). A soil lookup table with soil code and soil name was prepared to relate the map with the model.

#### Land use

**Fig. 3:**
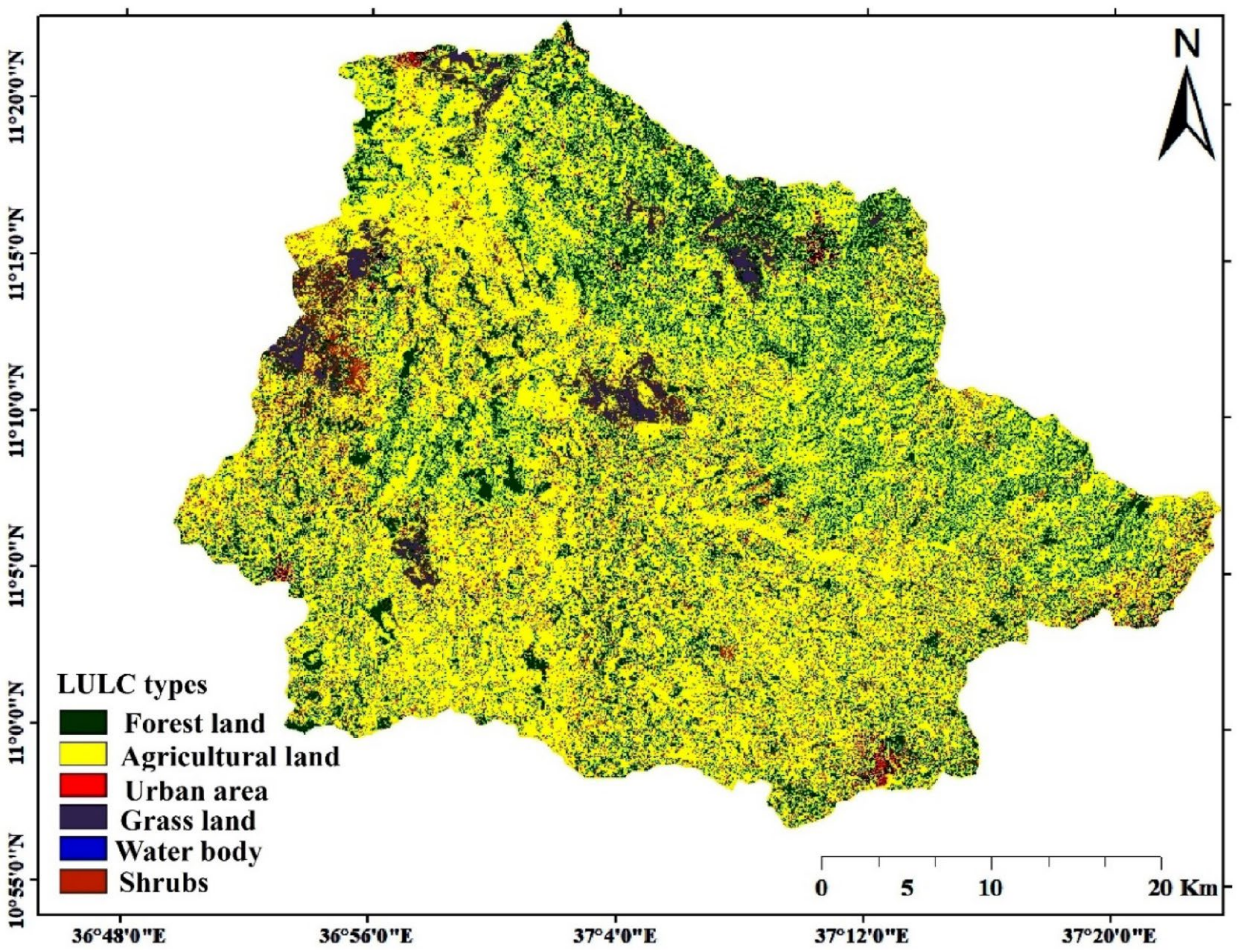
Land use and land cover map. The map was produced using ArcGIS Pro (version 3.0.3, ESRI; https://www.esri.com/en-us/arcgis/products/arcgis-pro).

The SWAT model heavily depends on land use as an input, which has significant effects on the definition and behavior of hydrological response units (HRUs). Land use land cover classification was conducted using ArcGIS Pro. World imagery of 2020 with 1.19 m resolution was extracted using the watershed boundary as a mask layer. Six land use classes and a new schema were generated using the extracted imagery under the imagery tab in the ArcGIS Pro training sample manager. Schema and training sample points were saved. After saving the classification schema and training points, the extracted imagery was selected. Under the imagery tab in the classification tools, the ‘Classify Images’ option was used, and the Maximum Likelihood Classification algorithm was applied to classify the image. The training samples were added, and the classification was performed by specifying the output directory^21^. After classifying the image small errors in the classification results were edited to individual features or objects by creating a polygon on the classified image and replacing the first land use type with the new land use types using a reclassifier in the classification tool (Fig. 3). After the accurate land use classification, a lookup table was prepared for each land use and land cover type having four-letter codes recommended by SWAT to make the land use name compatible with the model. Six land uses were identified (Table 3).

**Table 3:** Land use and SWAT code.

Land use	SWAT code	Area (ha)	Percent
Forest land	FRST	34938.76	21.32
Agricultural land	AGRL	110127.03	67.20
Urban area	URBN	1372.94	0.84
Grass land	GRAS	4993.14	3.05
Water body	WATR	537.5	0.33
Shrubs	SHRB	11900.24	7.26

#### Slope

**Fig. 4:**
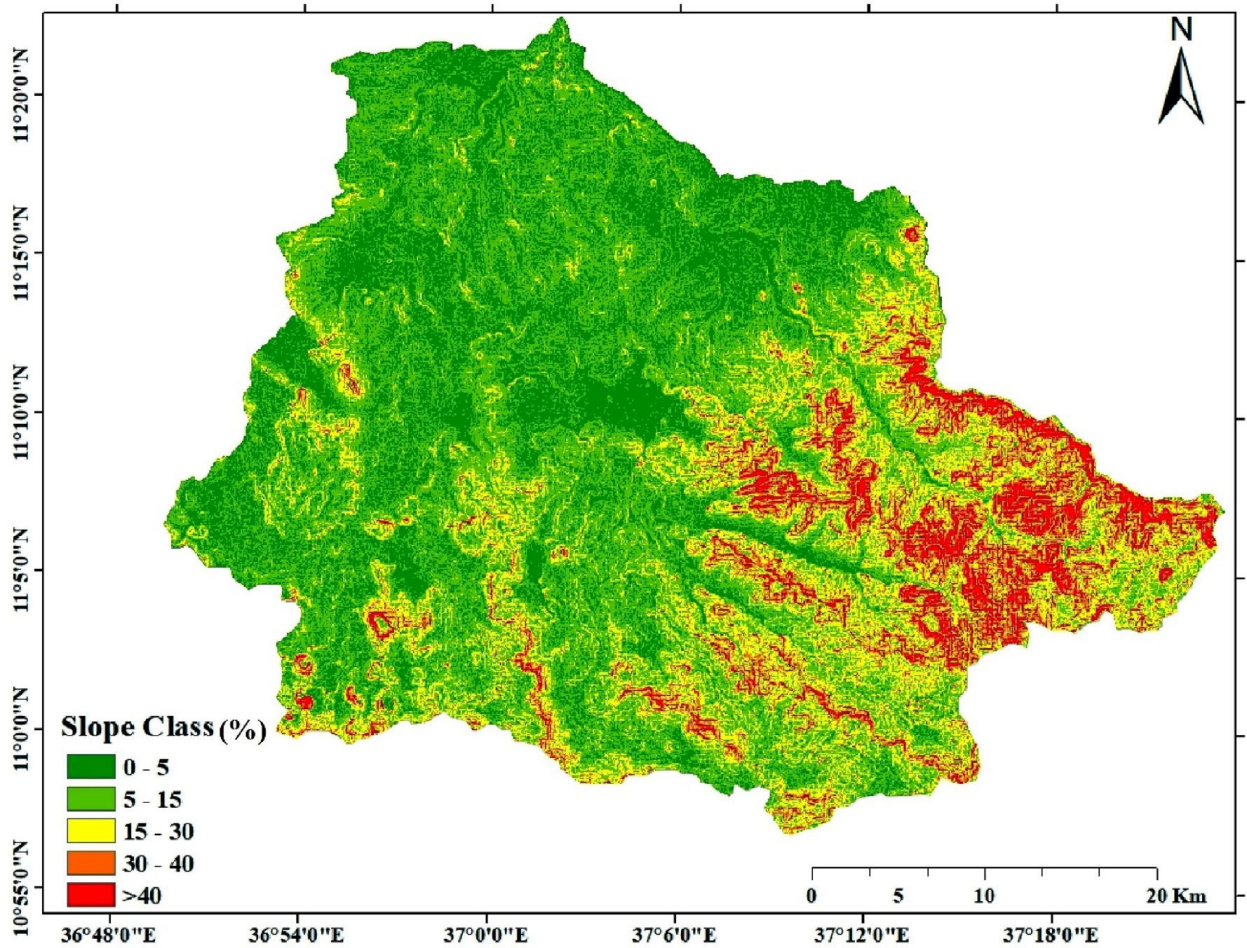
Slope map. The map was produced using ArcGIS (Version 10.8.2, https://www.esri.com/en-us/arcgis/products/arcgis10.8.2).

The slope parameter used in this study was derived from a SRTM 1 Arc-Second Global DEM using ArcGIS software. Specifically, the “Slope” tool available under the Spatial Analyst extension was employed to compute the slope within the study area. The resulting slope raster was subsequently utilized as one of the key input layers for the soil erosion and sediment yield modeling. The slope classes were determined based on commonly accepted terrain classification standards used in land evaluation and watershed management, following Morgan^44^. The catchment was divided into five slope classes: 0–5% (flat), 5–15% (smooth to undulating), 15–30% (steep/undulating), 30–40% (moderately steep), and > 40% (steep slopes). This classification aligns with categorizations applied in hydrological models and erosion studies for hilly and mountainous regions, ensuring a structured approach to terrain assessment (Fig. 4).

#### Hydrological data

The calibration and validation of the simulated results were performed using sediment and streamflow data recorded at the catchment outlet. The daily streamflow data, provided by the Ministry of Water and Energy, Ethiopia, covered the period from 2002 to 2021. In cases where continuous sediment data were unavailable, the sediment rating curve was used to generate time-step data^17,21,45,46^. The continuous daily time step sediment yield was calculated by using the sediment rating curve equation Eq. 3^48^.

3 $$\:{Q}_{s}=\:{a\times\:Q}^{b}.$$

where Qs is sediment load (ton/day) and Q is streamflow (m^3^/s), a and b are empirical constants determined by regression analysis.

**Fig. 5:**
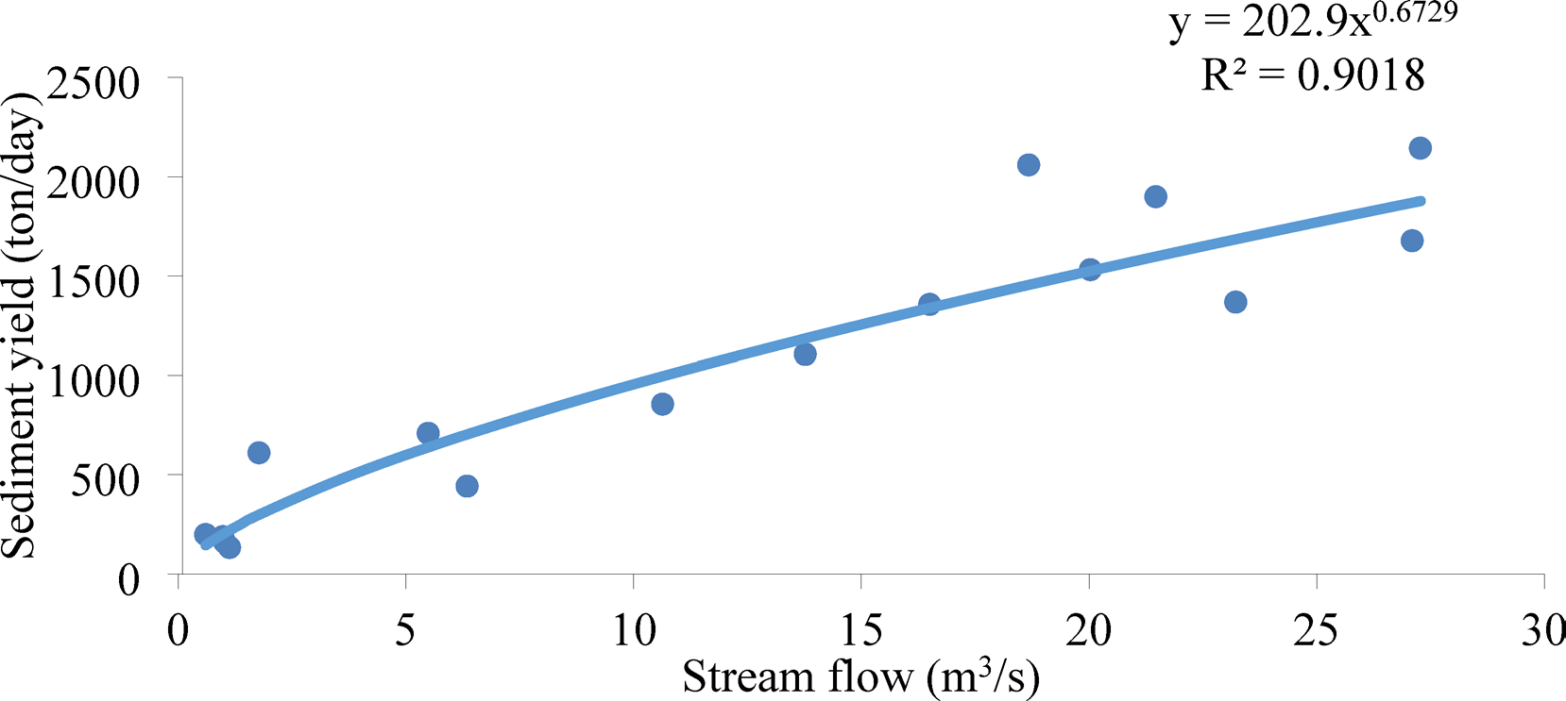
Sediment rating curve.

To estimate daily sediment yield from streamflow data, a sediment yield rating curve was constructed using the available sediment concentration records. Due to the discontinuous nature of the sediment data, a discharge-sediment rating curve with an R² of 0.90 was developed. A power equation was applied to establish a logarithmic relationship between discharge and sediment yield (Fig. 5). Similar methodologies have been successfully implemented in other Ethiopian watersheds, including the Andasa watershed^47^ and Ribb Reservoir Eq. (4)^48^.

4 $$\:{Q}_{s}=\:{202.9\times\:Q}^{0.6729}.$$

where Qs is sediment load (ton/day) and Q is streamflow (m3 /s).

### Model setup

**Fig. 6:**
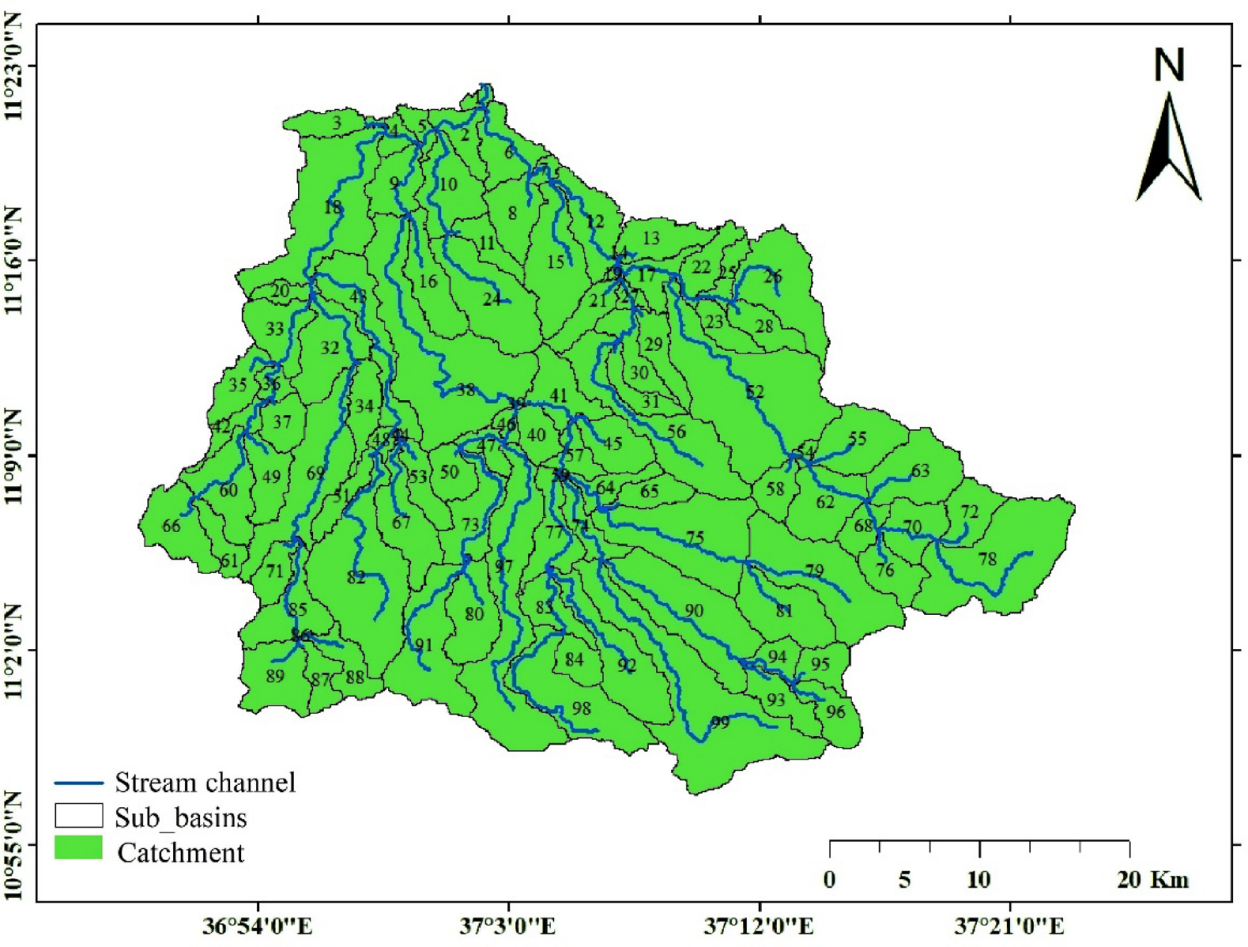
Sub-catchments. The map was produced using ArcGIS (Version 10.8.2).

SWAT + version 2.1.1 of the QGIS interface, QSWAT + was used to perform the analysis. The first procedure in a simulation of the SWAT model was watershed delineation. Projected SRTM 1 Arc-Second Global DEM data with 30 m resolution was used to delineate the catchment boundary. After generating flow direction and flow accumulation using the anticipated SRTM 1 Arc-Second Global DEM, SWAT automatically defined the streams. It then created the catchment boundary once the outlet and inlet definitions were provided^40^. The study area was divided into 99 smaller sub-basins, as shown in (Fig. 6). The characteristics of each sub-basin were then determined before proceeding with the Hydrological Response Unit (HRU) analysis. According to the Soil and Water Assessment Tool (SWAT), an HRU represents a unique combination of soil properties, land use, and slope characteristics that aims to decrease the variability of the landscape. Each sub-basin was separated into HRUs, resulting in a total of 99 HRUs, which means each sub-basin corresponds to one HRU. This ensures that both HRUs and sub-basins share the same boundaries but are characterized by their distinct environmental features, such as land use, soil properties, and slope characteristics. The thresholds of 20% slope, 10% soil, and 20% land use, as recommended by Winchell et al.^49^, were used to exclude minor land use, soil, and slope types. A total of 99 HRUs were generated. After the generation of HRU the climate data prepared in the SWAT input format for each station were imported into the model.

**Fig. 7:**
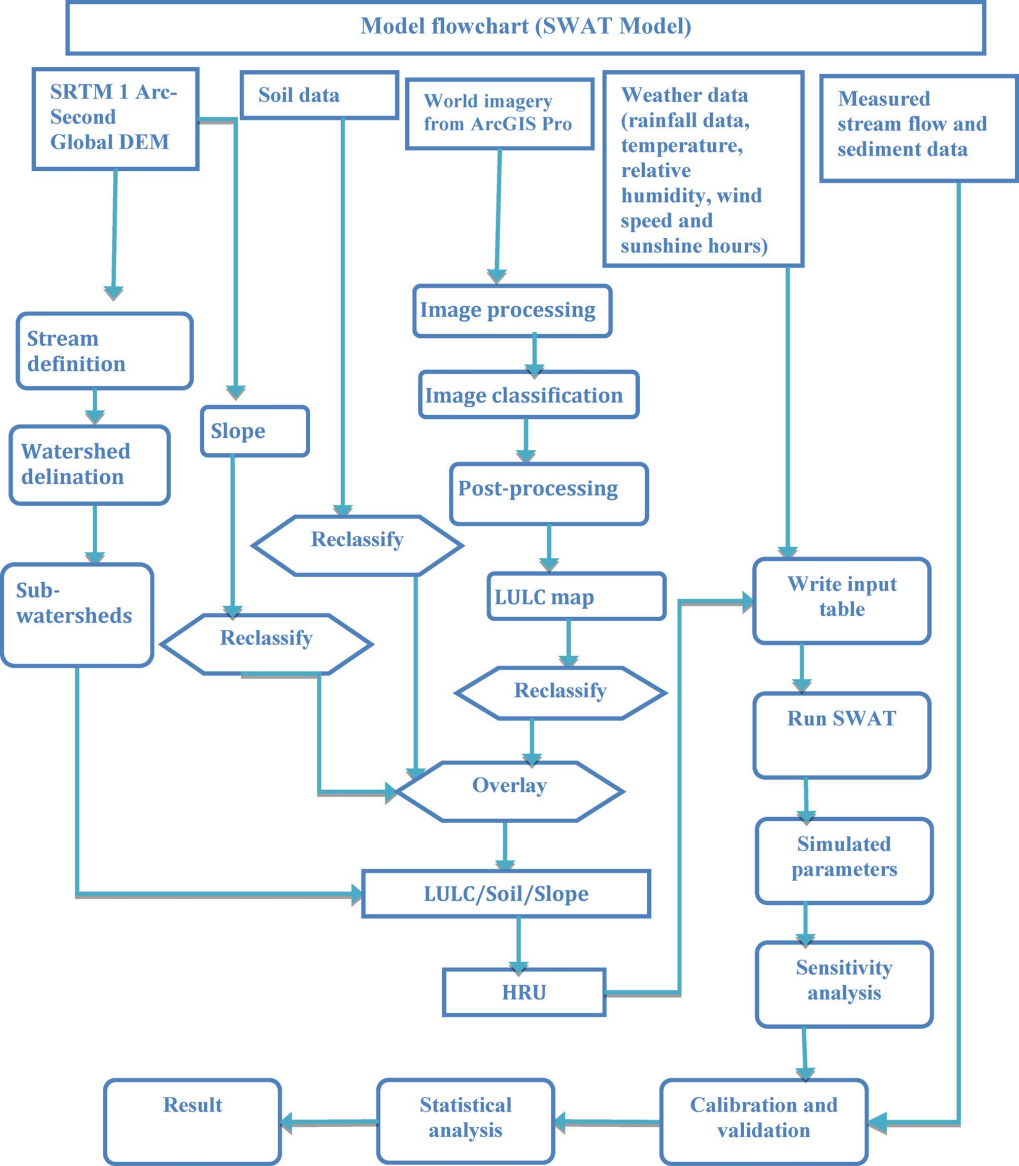
Model flowchart.

The upper Gilgel Abay catchment has been subdivided into 99 HRUs and sub-basins (Table 4). The catchment was divided into six primary land use categories and six key soil types. Agriculture makes up almost 67% of the watershed, while forest and shrubland make up about 21 and 7%, respectively. The primary soil type is Andosols accounting for 65%, followed by Vertisols (18%) and Leptosols (16%). The catchment’s slope is rated as gentle to moderate on 68% of the area, while 31% is classified as steep to extremely steep. Figure 7 clearly shows the general workflow of the study. Sensitivity analysis is an essential method for identifying parameters that have a substantial impact on model outputs^50^. It offers valuable insights into which parameters most influence the variation in outputs resulting from input variability^51^. Sensitivity analysis was performed separately for both streamflow and sediment yield to ensure a comprehensive evaluation of the model’s performance. Sensitivity analysis was conducted using the SWAT-CUP tool with the Sequential Uncertainty Fitting Algorithm Version 2 (SUFI-2). Following the sensitivity analysis, the model was calibrated and validated using the SWAT-CUP tool with the SUFI-2 algorithm. Calibration was based on daily observed streamflow and sediment data from the Gilgel Abay gauge station for the period 2002–2015, while validation was conducted for 2016–2021.

**Table 4:** HRUs report for the Gilgel Abay catchment.

Number of HRUs: Number of sub-basins: Watershed area(ha):	99 99 163,557.31 ha		
		Area (ha)	Area (%)
Land use type	Agriculture	109,852.7	67.16
	Forest land	34,966.9	21.38
	Shrubs	11,846.4	7.24
	Grasses	4988.7	3.05
	Settlements	1379.3	0.84
	Water body	523.3	0.32
Soil type	Andosols	106,582.6	65.16
	Vertisols	29,981.5	18.33
	Leptosols	26,450.1	16.17
	Luvisols	519.8	0.317
	Cambisols	21.8	0.013
Slope class (%)	0–5	44,439.6	27.2
	5–15	67,212.8	41.1
	15–30	32,930.9	20.1
	30–40	8804.3	5.4
	> 40	10164.8	6.2

### Model performance evaluation

The study assessed the SWAT model’s efficiency using Nash Sutcliffe efficiency (NSE) Eq. (5), coefficient of determination (R^2^) Eq. (6), percent bias (PBIAS) Eq. (7) and RMSE Eq. (8)^52^.

5 $$\:\text{N}\text{S}\text{E}=1-\left[\frac{{\sum\:}_{\varvec{i}=1}^{\varvec{n}}{({\varvec{X}}_{\varvec{i}}-{\varvec{Y}}_{\varvec{i}})}^{2}}{{\sum\:}_{\varvec{i}=1}^{\varvec{n}}{({\varvec{X}}_{\varvec{i}}-\stackrel{-}{\varvec{X}})}^{2}}\right].$$

Where *Xi* represents the measured values, *Yi* the simulated values, and $\:\stackrel{-}{X\:}$the average measured value.

6 $$\:\text{R}2=\frac{{\left[{\sum\:}_{\varvec{i}=1}^{\varvec{n}}({\varvec{X}}_{\varvec{i}}-\stackrel{-}{\varvec{X}})({\varvec{Y}}_{\varvec{i}}-\stackrel{-}{\varvec{Y}})\right]}^{2}}{\sqrt{\sum\:_{\varvec{i}=1}^{\varvec{n}}\left[({{\varvec{X}}_{\varvec{i}}-\stackrel{-}{\varvec{X}})}^{2}\sum\:({{\varvec{Y}}_{\varvec{i}}-\stackrel{-}{\varvec{Y}})}^{2}\right]}}.$$

Where *Xi* represents the measured values, *Yi* the simulated values,$\:\:\stackrel{-}{X}$ the average measured value and $\:\stackrel{-}{Y}$ the average simulated value.

7 $$\:\text{P}\text{e}\text{r}\text{c}\text{e}\text{n}\text{t}\:\text{b}\text{i}\text{a}\text{s}\:\left(\text{P}\text{B}\text{I}\text{A}\text{S}\right)=\left[\frac{{\sum\:}_{=i}^{n}{(O}_{i}-{P}_{i\:})\:\times\:100}{{\sum\:}_{=i}^{n}\left({O}_{i}\right)}\right].$$

Where $\:{O}_{i}\:$represents $\:{i}^{th}$ observed value, $\:{P}_{i}$ the $\:{i}^{th}$ simulated value.

RMSE estimates the average error magnitude between simulated and observed values. It gives higher weight to larger errors due to squaring the differences, making it especially sensitive to outliers (53).

8 $$\:\text{R}\text{o}\text{o}\text{t}\:\text{M}\text{e}\text{a}\text{n}\:\text{S}\text{q}\text{u}\text{a}\text{r}\text{e}\:\text{E}\text{r}\text{r}\text{o}\text{r}\:\left(\text{R}\text{M}\text{S}\text{E}\right)=\sqrt{\frac{1}{n}\sum\:_{i=1}^{n}{({Q}_{i}-{S}_{i})}^{2}}.$$

Where $\:{Q}_{i}$ represents observed value, $\:{S}_{i}$ simulated value

According to Moriasi et al.^53^, the value of the ranges of the model performance evaluation parameters is described below in (Table 5).

**Table 5:** Values of model performance evaluation coefficients. (Source: Moriasi et al.^53^).

*R* ^2^	NSE	PBIAS		RMSE	Performance rating
		Sediment	Flow		
0.7 < R^2^ < 1	0.75 < NSE < 1	< 15%	< 10%	0 < RMSE ≤ 0.50	Very good
0.6 < R^2^ < 0.7	0.65 < NSE < 0.75	15–30%	10% < PBIAS < 15%	0.5 < RMSE ≤ 0.60	Good
0.5 < R^2^ < 6	0.5 < NSE < 0.65	30–55%	15% < PBIAS < 25%	0.6 < RMSE ≤ 0.70	Satisfactory
R^2^ < 0.5	NSE < 0.5	> 55%	> 25%	RMSE > 0.70	Unsatisfactory

## Results and discussion

### Sensitivity analysis

Initially, a set of parameters were selected based on recommendations from previous studies^42,54–56^. Sensitivity analysis was conducted using the SWAT-CUP tool, employing the Sequential Uncertainty Fitting Algorithm Version 2 (SUFI-2). In line with Yang et al.^57^, multiple iterations were performed to ensure the robustness and reliability of the optimized parameter values. As a result, several parameters were identified as sensitive, showing significant influence on the model outputs. Sensitivity analysis was carried out separately for both streamflow and sediment yield, and the most responsive parameters along with their corresponding t-stat and p-values are presented in (Table 6). The t-statistic values are used to assess the sensitivity of the parameters, with larger absolute t-stat values indicating greater sensitivity. This means that parameters with higher absolute t-stat values have a more substantial influence on the model’s output. On the other hand, p-values are used to determine the statistical significance of the sensitivity. A parameter is considered significant if its p-value is close to 0, indicating that the observed effect is unlikely due to random chance and that the parameter significantly affects the model’s behavior^58^. In the SWAT model calibration process, particularly when using tools like SWAT-CUP, specific qualifiers such as v_, r_, and a_ are used to define how model parameters are adjusted. The prefix v_ indicates that the existing parameter value will be replaced by a new value within a specified range. The r_ qualifier signifies a relative change, meaning the parameter value is adjusted by a specified percentage of its original value. This is useful for scaling parameters proportionally. In contrast, the a_ qualifier denotes an absolute change, where a fixed value is added to or subtracted from the original parameter value. These qualifiers offer flexibility in the calibration process while ensuring parameter changes remain within realistic and meaningful bounds Abbaspour^59^.

**Table 6:** Sensitive parameters with their t-statistic and p-values.

Hydrological parameters	Parameter_name	t-statistic	*p*-value	Rank
Stream flow	1: R__CN2.mgt	3.5	0.001	1
	2: R__ALPHA_BF.gw	2.1	0.04	2
	3: R__GW_REVAP.gw	1.5	0.15	3
	4: R__CH_N2.rte	0.8	0.45	4
	5: R__RCHRG_DP.gw	2.8	0.01	5
	6: R__SLSUBBSN.hru	1.2	0.22	6
	7: R__SOL_K(.).sol	0.5	0.60	7
	8: R__REVAPMN.gw	1.8	0.07	8
	9: R__OV_N.hru	0.3	0.75	9
	10: R__GWQMN.gw	2.5	0.02	10
	11: R__ALPHA_BNK.rte	1.0	0.30	11
Sediment yield	12: USLE_C	6.9	0.01	1
	13: SPCON	5.9	0.03	2
	14: USLE_P	4.8	0.08	3
	15: CH_COV1.rte	3.1	0.09	4
	16: CH_ERODMO.rte	2.6	0.02	5
	17: SPEXP	3.7	0.15	6

### Calibration and validation of streamflow

**Fig. 8:**
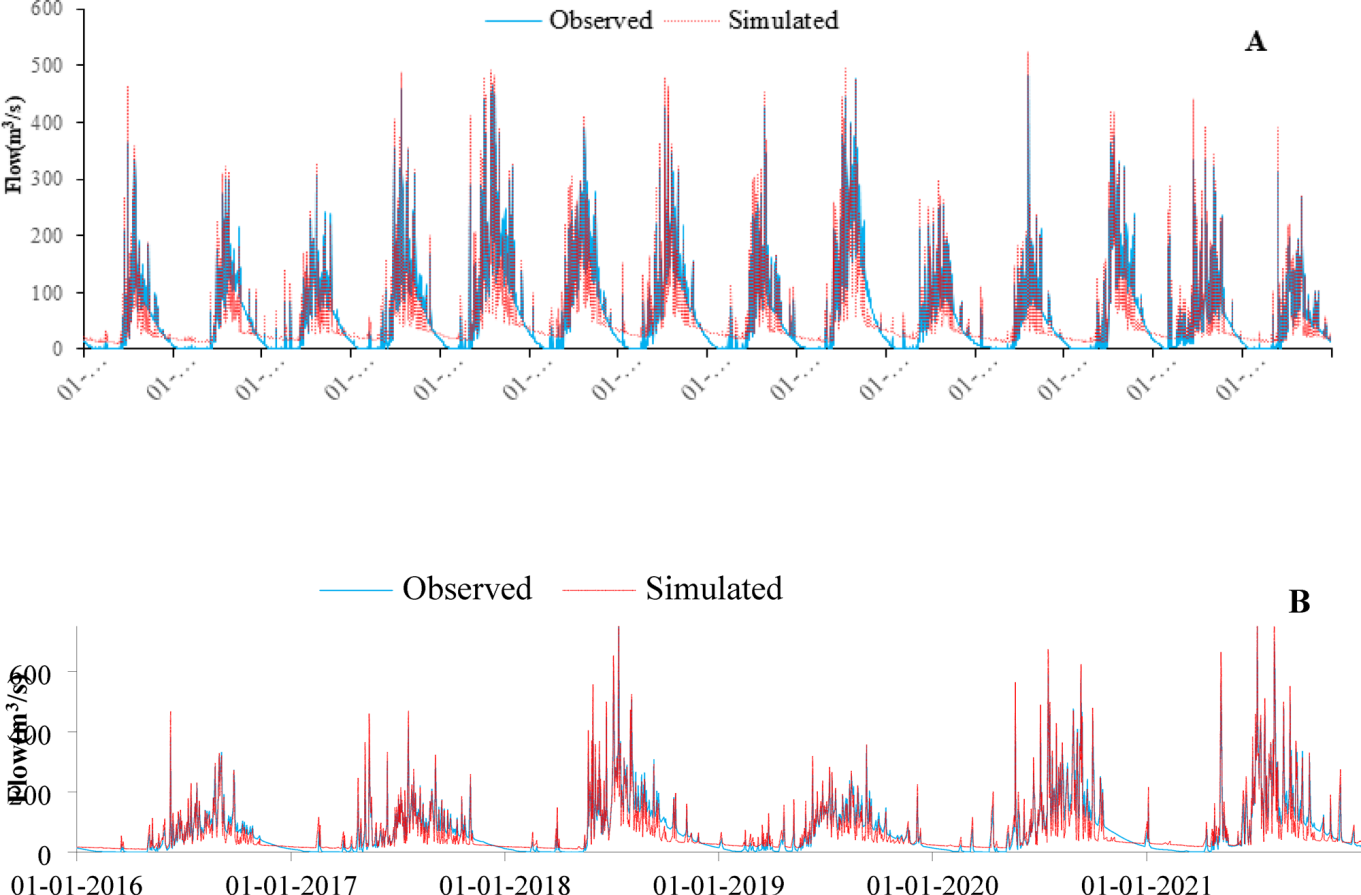
Daily stream flow calibration (**A**), and validation (**B**).

The model was calibrated using the SWAT-CUP tool, specifically employing the SUFI-2 algorithm. After identifying sensitive parameters, calibration was performed based on daily observed stream flow and sediment data at the Gilgel Abay gauge station. Calibration was performed from the year 2002 to 2015 (Fig. 8A) and validation was done from the year 2016 to 2021 (Fig. 8B). According to Moriasi et al.^53^. , the value of model performance evaluation criteria was in the acceptable range in both calibration and validation of stream flow (Table 7).

**Table 7:** Model performance assessment criteria during stream flow calibration and validation.

Stations	Calibration				Validation			
	*P*-Factor	*R*-Factor	*R* ^2^	NSE	*P*-Factor	*R*-Factor	*R* ^2^	NSE
Gilgel Abay	0.75	0.78	0.9	0.9	0.79	0.8	0.9	0.91

### Calibration and validation of sediment

**Fig. 9:**
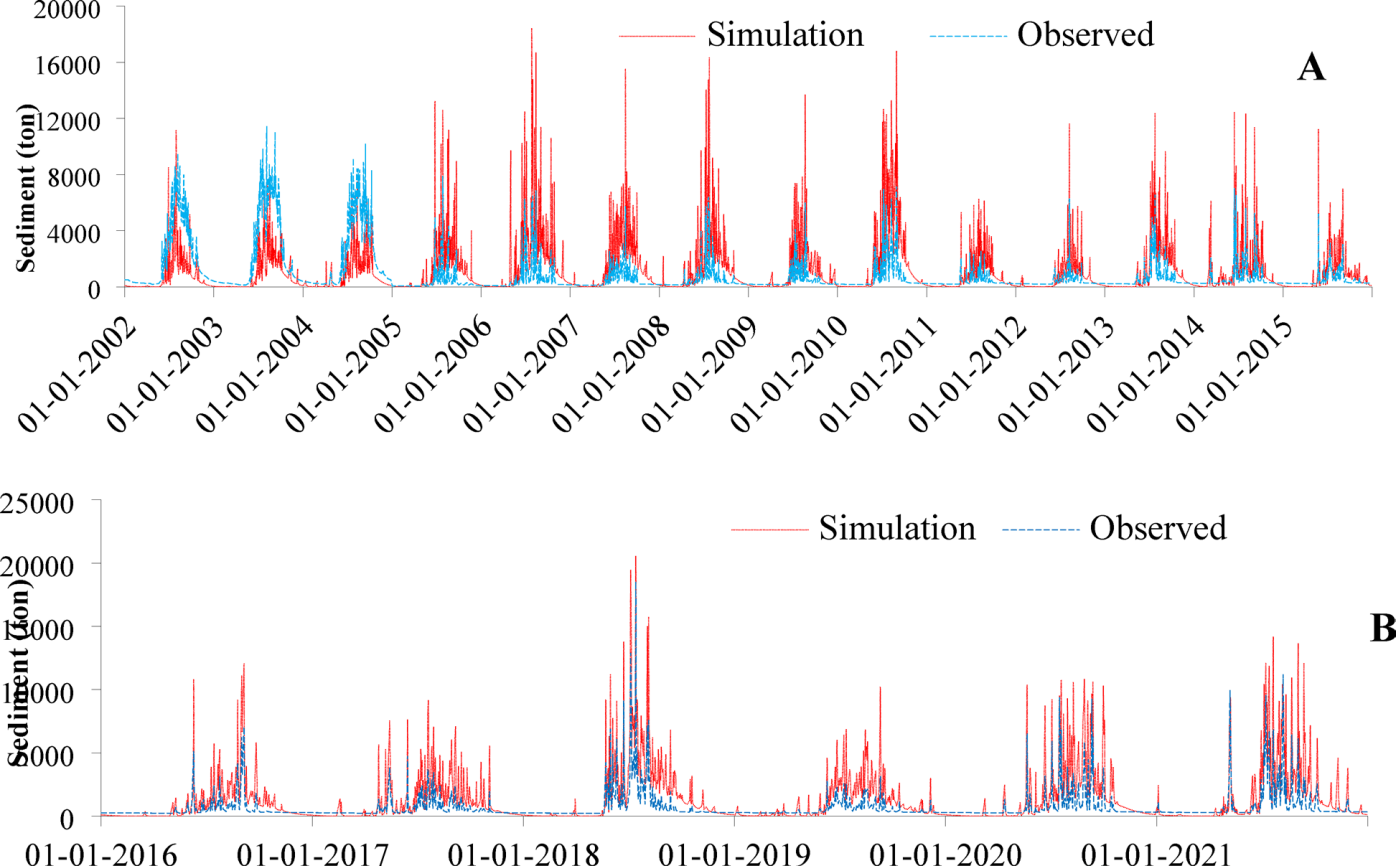
Daily sediment calibration (**A**), and validation (**B**).

Since runoff and stream flow are the main factors affecting sediment transportation, the daily simulated sediment was calibrated for the period from 2002 to 2015 after the calibration and validation of stream flow (Fig. 9A). Validation of sediment was performed for the period from 2016 to 2021 (Fig. 9B). According to Moriasi et al.^53^. , the values of model performance evaluation criteria are in the acceptable ranges for both the calibration and the validation of sediment (Table 8).

**Table 8:** Model performance assessment criteria during sediment calibration and validation.

	Calibration				Validation			
	*P*-Factor	*R*-Factor	*R* ^2^	NSE	*P*-Factor	*R*-Factor	*R* ^2^	NSE
Gilgel Abay	0.75	0.7	0.69	0.63	0.79	0.72	0.67	0.66

### Soil erosion risk extent

**Fig. 10:**
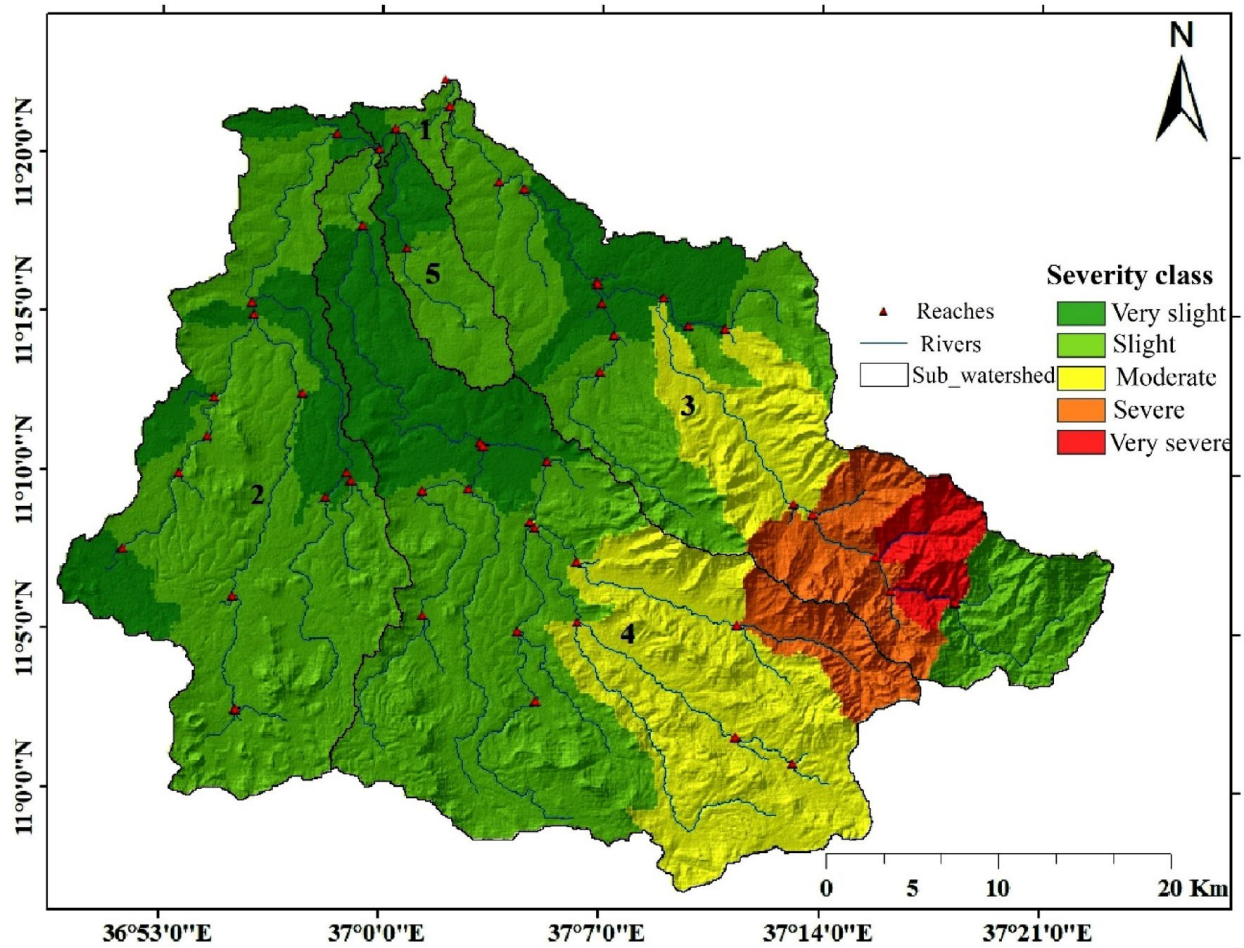
Soil erosion severity map. The map was produced using ArcGIS (Version 10.8.2, https://www.esri.com/en-us/arcgis/products/arcgis10.8.2).

The predicted annual soil loss in the catchment area is estimated to be 9510.3 tons/ha. The estimated mean annual values of soil loss varied between 0.15 tons ha^-1^ year^-1^ in the relatively flat northern areas to steep slope portions exceeding 53.88 tons ha^-1^ year^-1^. The average yearly loss of soil from the basin is 86,415,499 tons. The generated average annual soil loss is grouped into very severe, severe, moderate, slight and very slight^60–62^ (Fig. 10). The classification shows that 13876.97 ha, which covers 8.48% of the total catchment, was classified under the very severe soil erosion class. Further, a total of 24487.5 ha, which represents 14.97% of the catchment, was classified into the severe level of the soil erosion classification. The moderate level of severity class was assigned to 19.94% of the catchment area. The remaining 42.5% and 14.1% are under the slight and very slight levels of soil erosion, respectively (Table 9). Thus, the results revealed that 38364.47 ha of the catchment are in the very severe and severe erosion classes, covering 23.45% of the total catchment. Such soil loss rates have been recorded in places of somewhat steep terrain as well as in regions where the original forest cover has been converted to agricultural land and degraded bushland. The largest part of the catchment, covering 67.2% of the total area, is agricultural land and the farmers are relying on crops. The sub-watersheds within the study area are labeled numerically on the map to improve clarity. The points displayed in Fig. 10 correspond to the outlets of the river reaches within the catchment. These reaches play a critical role in the routing of water, and sediment^33^.

The upper part of the Upper Gilgel Abay catchment is significantly affected by soil erosion, particularly on agricultural land. This is driven by intensive farming practices, deforestation, poor land management, and the conversion of forests into croplands. Additional contributors include over-cultivation, overgrazing, farming on steep slopes, and forest fires caused by human activity. These factors collectively degrade soil fertility, cause reservoir siltation, and reduce agricultural productivity. The area’s rugged and undulating topography further exacerbates erosion, placing much of the catchment under severe to very severe erosion levels. Based on the spatial distribution of erosion severity and the identified driving factors, appropriate soil and water conservation measures should be implemented such as contour farming, agroforestry, and conservation tillage; restoring degraded forest areas through reforestation; restricting agricultural activities on steep slopes; and introducing controlled grazing systems to prevent overgrazing.

Fenta et al.^63^. , found that yearly soil loss in Ethiopia’s major river basins was estimated to be 1.9 billion tons/year, and sediment yield was 410 million tons/year. The rate of soil erosion exceeds the acceptance level change with the basin characteristics, causes reservoir siltation, and affects human beings’ lives^64,65^.

In contrast to previous reports conducted in Ethiopia, the mean annual soil loss rate estimated at the northwestern highland of Ethiopia by Zeleke^66^ which is 243 t ha^-1^ year^-1^ was much higher than this result; and this result is also smaller than 93 ton ha^-1^ year^-1^ found at Chemoga watershed by Bewket and Teferi^26^, 84 t ha^-1^ year^-1^ estimated at northwestern Ethiopia by Selassie and Belay^67^, and in line with those reported as 47.4 t ha^-1^ year^-1^ at Koga watershed by Gelagay and Minale^27^. In Africa, due to the rivers’ capacity to flow massive volumes of water, soil erosion occurs frequently and to a great extent^68^. Setegni et al.^69^. , discovered that in the lake Tana sub-basins, 18.4% of the catchment had sediment outputs greater than 30 tons/ha, considered areas with considerable erosion potential. The land use map of the research area revealed a decline in forest cover, primarily driven by the expansion of agricultural activities. This reduction in forest cover has contributed to increased soil erosion. Soil erosion rate also varies with the vegetation cover found in the area^70^. The most significant amount of soil loss was generated from agricultural land (9412.32 t/ha) which was followed by grassland (35.55 t/ha). In terms, of soil types, the greatest amount is estimated in the area having Luvisols with a value of (7068.66 t/ha) followed by Leptosols with soil loss of 1512.15 t/ha/yr. The lowest amount of soil loss contribution is obtained from Cambisols (97.11 t/ha/yr) (Table 10).

Sub-basins are labeled from 1 to 5 on the map. Sub-basins 3 and 4 are the primary contributors to soil erosion in the area, as they contain moderate and severe erosion severity classes. In contrast, Sub-basins 1, 2, and 5 contribute negligibly to the overall soil erosion. Sub-basin 1 is predominantly classified in the very slight erosion category (dark green), resulting in a minimal contribution to soil erosion due to the prevalence of low-severity areas. Similarly, Sub-basin 2 is mainly in the very slight erosion category (dark green), which leads to a minimal impact on soil erosion in the region. Sub-basin 5, like Sub-basin 1, is largely in the very slight erosion category (dark green), contributing very little to the overall soil erosion.

The spatial variation of soil erosion risk is affected by different variables such as the characteristics of soil, vegetative cover, topography, climate, and human activities. The amount of organic matter present in the soil and the structural arrangement of the soil affects the resistance of the soil to rainfall^71^. According to recent studies, greater rates of erosion have been caused by overgrazing, deforestation, and inappropriate farming practices^72^. Higher surface runoff and soil loss are closely associated with an absence of plant cover^73^. Soil degradation is still mostly caused by inappropriate land management techniques, such as over-tilling, monocropping, and ineffective water management^71^.

**Table 9:** Erosion severity class.

Soil loss rate (ton/ha/year)	Severity class	Area (ha)	% Area	Total annual soil loss (ton)	Soil loss (%)
< 5	Very slight	23063.31	14.1	5812354.269	6.73
5–15	Slight	69512.35	42.5	50049656.64	57.92
15–30	Moderate	32617.19	19.94	14766528.28	17.09
30–50	Severe	24487.5	14.97	11472611.67	13.27
> 50	Very severe	13876.97	8.48	4314348.72	4.99
Total		163557.31	100.00	86,415,499.5	100.00

Source: Haregeweyn et al.^57^. and Yesuph and Dagnew^58^.

**Table 10:** Soil type contributions to soil loss.

Soil type	Area (%)	Annual soil loss contribution (t/ha)
Luvisols	65.16	7068.66
Leptosols	0.317	1512.15
Vertisols	18.33	734.4
Cambisols	0.013	97.11
Andosols	16.13	97.98

### Sediment yield distribution

According to the result of the model, at the catchment’s outlet, the greatest and lowest annual sediment yields were 318, 233 tons, and 61,575 tons, respectively. The average sediment yield at the main outlet is 184,695 tons. Since some of the eroded sediment particles inside the catchment get stuck and deposited in the upstream reaches, all sediment particles within the catchment are not transported to the outlet.

This soil loss may cause a serious onsite impact on the catchment and also offsite impacts. The off-site impact of soil erosion is mainly related to sedimentation in the water bodies. Both on- and off-site effects of the eroded soil materials are harmful to both plants and water^74^. All ecosystem needs water to function properly on qualitative as well as quantitative levels. Ecosystems are severely harmed by both declining water availability and deteriorating water quality^75^.

According to Andualem^76^, about 1,018,000 tons of sediment flow into the lake Tana basin which is estimated by using the recent land use in the tributaries. This value reduces the capacity of the lake and the capacity of the channels, which causes the reduced volume of lakes and rivers.

Since the upper Gilgel Abay catchment is one of the major tributaries of the lake Tana basin, the higher sediment yield in this catchment results in sedimentation and deterioration of the water quality. The transporting agents don’t contain only soil they may leach nitrogen, phosphorus and other chemicals in fertilizers applied in the agricultural field when soil erosion occurs. This may increase the growth of algae and other species like water hyacinth invasion such as Eichhornia Crassipes (Enbochi) which reduces the concentration of oxygen in the water and aquatic animals will die because of insufficient oxygen and the water body will also dry.

### Sediment yield trend analysis

Trend analysis was conducted to assess the sediment production over time. The catchment was divided into 99 sub-catchments during the model setup phase (Fig. 6). For the trend analysis, four sub-basins were selected. These sub-basins were chosen based on their representativeness of varying characteristics (e.g., land use, soil types, and topography) and the availability of consistent historical sediment yield data. The selected sub-basins, which are associated with their IDs in Fig. 6, are situated in the northern part of the catchment, as shown on the map. These areas experienced significant land use changes, including agricultural expansion and deforestation, making them suitable for analyzing sediment yield trends. The selection was explicitly based on the availability of historical sediment data at these outlets, which was collected from the gauge station and sediment-related observations documented in previous studies^17,77^.

**Fig. 11:**
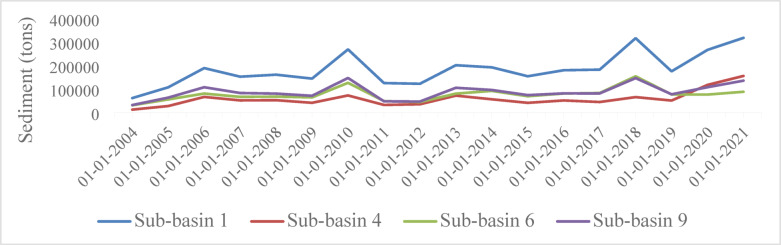
Sediment yield trend analysis.

During the summer months, higher sediment yields were observed due to heavy rainfall and increased surface runoff. To provide a clearer understanding of sediment yield distribution and trends, graph and table are included. Figure 11; Table 11 presents the annual sediment yields for the selected sub-basins, along with the trends observed over the years. These additional visual representations serve to enhance the interpretation of the sediment dynamics and provide a more comprehensive view of how sediment yields vary spatially within the study area. The trend of sediment yield over the years shows a general increase, with the maximum yield recorded in 2021 (318,233 tons), followed by 316,869 tons in 2018 (Fig. 11). This trend underscores the influence of land management practices on soil erosion dynamics. The dramatic increase in 2021 can be attributed to multiple factors, including the expansion of agricultural land, deforestation, overgrazing, intense rainfall events, and insufficient soil conservation measures. These variables collectively reduce soil stability and increase erosion rates, as supported by several studies^62,63^. The increased conversion of forested land to agricultural use, excessive grazing, the presence of heavy rains and inadequate soil conservation measures are some of the causes which contributed to the dramatic increase in sediment yield in 2021^17^. These variables collectively decrease soil stability and accelerate erosion rates^61^.

In general, the trend of sediment is increasing from year to year (Table 11), this might be due to the increment in population, deforestation, intensive agriculture, and the variability of climate in the catchment. Without upstream conservation measures, the catchment risks severe nutrient depletion in its topsoil, threatening food security for local farmers. Moreover, sedimentation in the lake Tana basin could lead to significant environmental and economic challenges, necessitating immediate intervention. A limitation of this study is the current quality and quantity of meteorological and hydrological data, which if enhanced, could greatly improve model calibration and validation for more accurate soil erosion and sediment transport assessments.

**Table 11:** Trend analysis of sediment yield.

Time series	First year	Last year	Test Z	Signific.	Q (tons)
Sub-basin 1	2004	2021	2.65	**	8519.2813
Sub-basin 4	2004	2021	1.97	*	2578.506
Sub-basin 6	2004	2021	2.20	*	1717.09
Sub-basin 9	2004	2021	1.59		582.49

## Conclusions

This study modeled sediment yield and soil erosion in the Upper Gilgel Abay catchment, Ethiopia, using the SWAT model. The results reveal significant soil erosion risk, with an estimated annual soil loss of 9,510.3 tons/ha, leading to an average yearly loss of 86,415,499 tons from the basin. Severe erosion affects 23.45% of the catchment area, threatening land productivity and food security, particularly in agricultural lands on steep slopes with inadequate vegetation cover. Sediment yield at the catchment outlet averages 184,695 tons, with downstream impacts on Lake Tana, including siltation and the spread of invasive water hyacinths.

To mitigate these issues, physical conservation measures, such as contour farming, ditches, and terracing, are essential. Future research should focus on evaluating the effectiveness of Best Management Practices (BMPs) to reduce soil erosion and sedimentation. Additionally, incorporating the impacts of land use changes and climate change into the modeling framework will be crucial for understanding how these factors influence soil erosion dynamics and sediment transport over time. Enhancing the quality and quantity of meteorological and hydrological data will also improve model accuracy, providing more precise assessments of soil erosion and sediment yield under varying conditions.

## Data Availability

The corresponding author can provide the data described in this study upon request.
